# Stromule Geometry Allows Optimal Spatial Regulation of Organelle Interactions in the Quasi-2D Cytoplasm

**DOI:** 10.1093/pcp/pcad098

**Published:** 2023-09-02

**Authors:** Jessica Lee Erickson, Jennifer Prautsch, Frisine Reynvoet, Frederik Niemeyer, Gerd Hause, Iain G Johnston, Martin Harmut Schattat

**Affiliations:** Department of Plant Physiology, Martin-Luther-University Halle-Wittenberg, Weinbergweg 10, Halle 06120, Germany; Department of Biochemistry of Plant Interactions, Leibniz Institute for Plant Biochemistry, Weinbergweg 10, Halle 06120, Germany; Department of Plant Physiology, Martin-Luther-University Halle-Wittenberg, Weinbergweg 10, Halle 06120, Germany; Department of Plant Physiology, Martin-Luther-University Halle-Wittenberg, Weinbergweg 10, Halle 06120, Germany; Department of Plant Physiology, Martin-Luther-University Halle-Wittenberg, Weinbergweg 10, Halle 06120, Germany; Department of Plant Physiology, Martin-Luther-University Halle-Wittenberg, Weinbergweg 10, Halle 06120, Germany; Department of Mathematics, University of Bergen, Realfagbygget, Bergen, Vestland 5007, Norway; Computational Biology Unit, University of Bergen, Høyteknologisenteret, Bergen, Vestland 5006, Norway; Department of Plant Physiology, Martin-Luther-University Halle-Wittenberg, Weinbergweg 10, Halle 06120, Germany

**Keywords:** Biotic stress, Optimal structures, Organelle interactions, Plastids, Stromules, *Arabidopsis thaliana*, *Nicotiana benthamiana*

## Abstract

In plant cells, plastids form elongated extensions called stromules, the regulation and purposes of which remain unclear. Here, we quantitatively explore how different stromule structures serve to enhance the ability of a plastid to interact with other organelles: increasing the effective space for interaction and biomolecular exchange between organelles. Interestingly, electron microscopy and confocal imaging showed that the cytoplasm in *Arabidopsis thaliana* and *Nicotiana benthamiana* epidermal cells is extremely thin (around 100 nm in regions without organelles), meaning that inter-organelle interactions effectively take place in 2D. We combine these imaging modalities with mathematical modeling and new in planta experiments to demonstrate how different stromule varieties (single or multiple, linear or branching) could be employed to optimize different aspects of inter-organelle interaction capacity in this 2D space. We found that stromule formation and branching provide a proportionally higher benefit to interaction capacity in 2D than in 3D. Additionally, this benefit depends on optimal plastid spacing. We hypothesize that cells can promote the formation of different stromule architectures in the quasi-2D cytoplasm to optimize their interaction interface to meet specific requirements. These results provide new insight into the mechanisms underlying the transition from low to high stromule numbers, the consequences for interaction with smaller organelles, how plastid access and plastid to nucleus signaling are balanced and the impact of plastid density on organelle interaction.

## Introduction

The process of enclosing the cellular lumen within membrane-bound compartments (compartmentalization) was crucial for cellular evolution and has led to a sophisticated and dynamic internal organization found in today’s modern eukaryotic cells (Gabaldon and Pittis 2015, Johnston 2019). These membrane-bound compartments, or organelles, enable the coexistence of multiple biochemical environments within a single cell. As a result, biochemical reactions can run under optimal conditions and opposing reactions can exist in a single cell. However, as a consequence of compartmentalization, many biochemical pathways are sequestered into two or more organelles (Lunn 2007). An example of such a sequestered pathway in plant cells is photorespiration, which involves reactions in plastids, peroxisomes and mitochondria (Eisenhut et al. 2019).

One consequence of creating membrane-bound organelles is that the sequestered compartments unavoidably obtain a shape, which is defined by their surrounding membranes. Interestingly, organelles often diverge from a simple spherical shape and undergo controlled morphological changes in response to external stressors or specific developmental processes (Sheahan et al. 2004, 2005, Fenton et al. 2021, Mathur 2021). It has been found that the shape of a membrane-bound compartment can impact its biochemical performance (Lizana et al. 2008) and that the location and dynamics of organelles influence their function and capacity for exchange and interaction (Chustecki et al. 2021, Giannakis et al. 2022). This suggests that the shape of organelles is not random but instead affords control over organelle function (Lizana et al. 2008, 2009).

An essential and ubiquitous instance of this structure–function relationship is found in plastids, organelles central to bioenergetics and metabolism in phototrophs. Plastids can exist in multiple specialized forms (Sierra et al. 2023), of which the chloroplast is among those responsible for light-driven carbon fixation (Choi et al. 2021). Plastids are not only characterized by their biochemical flexibility and involvement in multiple biochemical pathways (Weber and Fischer 2008, Brunkard et al. 2018, Sierra et al. 2023) but also known for their ability to change the shape of their enclosing membranes in drastic ways, by forming irregular shapes or long tubules (reviewed in Gray et al. 2001, Kwok and Hanson 2004c, Natesan et al. 2005). These thin, tubular extensions of the envelope membranes are filled with stroma and have therefore been named stromules (Kohler and Hanson 2000). Stromules can vary in length, ranging from a few microns to >65 µm, and have a thickness of about 0.4–0.8 µm. A notable characteristic of stromules is their dynamic nature, as they can rapidly extend, branch, kink and retract in just a few seconds or minutes (Gunning 2005). Despite being first observed over a century ago, stromules were largely ignored for years, as they are not easy to visualize under a light microscope (reviewed in Gray et al. 2001). However, with the development of fluorescence microscopy and the use of fluorescence proteins targeted to the stroma, stromules are now readily visible, and their prevalence throughout the *Viridiplantae* has been confirmed (Gray et al. 2011, Shaw and Gray 2011, Delfosse et al. 2015, Mathur 2021).

Despite their evolutionary conservation and controlled formation during stress and development (reviewed in Mathur 2021), stromule function is still not fully understood. Inspired by microscopic observations in different tissues, a number of functions have been proposed since their rediscovery in algae, in 1994 (Menzel 1994), and in vascular plants, in 1997 (Köhler et al. 1997). Different authors reported that peroxisomes and mitochondria come into close proximity with stromules, inspiring the hypothesis that stromules might increase the interactive surface of plastids, facilitating a more efficient exchange of metabolites (Kwok et al. 2004c and Kwok and Hanson 2004b, Schattat et al. 2011b, Barton et al. 2018) during photorespiration or inter-organelle stress-induced transmission of defense signals (Serrano et al. 2016) and modulation of reactive oxygen species (Pastor et al. 2013, Arnaud et al. 2017). Furthermore, observations describing stromules orientated toward nuclei, the cell periphery or plasmodesmata led authors to speculate that stromules connect distant parts of the cell with the plastid body without the need for plastid repositioning (Kwok et al. 2004c, Caplan et al. 2015). However, why some plastids extend stromules that are linear, some branched and some extend multiple stromules is not immediately addressed by this idea. Nor is it clear why plastids use stromule extension rather than motion to interact with distal partners.

Hypotheses regarding stromule function remain somewhat speculative, as the lack of stromule-specific mutants limits the capacity for direct experimental testing. Here, we propose an interdisciplinary approach that combines detailed microscopic measurements of stromules, plastids, and their cytoplasmic environment with mathematical and simulation modeling to evaluate how stromule formation impacts the interaction of plastids with their surroundings. We aim to use geometrical models of plastid and stromule structure, combined with optimization approaches, to explore how the outer structure of plastids may be controlled to facilitate optimal interactions between cellular components.

## Results

### The cytoplasm is extremely thin in epidermal cells, limiting organelle interactions in the quasi-2D region

#### Stromule extension might increase the interactive surface of plastids in restricted geometries.

In previous work imaging organelle dynamics and interactions (Chustecki et al. 2021, 2022), it was observed that the vacuole in *Arabidopsis* hypocotyl epidermal cells acts to compress the cytoplasm into a thin layer adjacent to the cell wall so that (for example) mitochondrial motion is often constrained to be largely planar (Chustecki et al. 2021). We reasoned that if this planarity restricts organelle motion and dimensionality, stromule extension and structure may facilitate more efficient exchange with different cellular regions than plastid motion. We therefore set out to identify the physical properties of the planar cytoplasm and plastids embedded within it. We focused on epidermal cells as a model system to allow for straightforward live imaging, without the high packing of organelles found in other tissues, but where stromules are known to form and interact with other compartments in response to different stimuli (Kwok and Hanson 2004b, Schattat et al. 2011b, Barton et al. 2018).

#### The cortical cytoplasm is a quasi-2D plane.

**Fig. 1:**
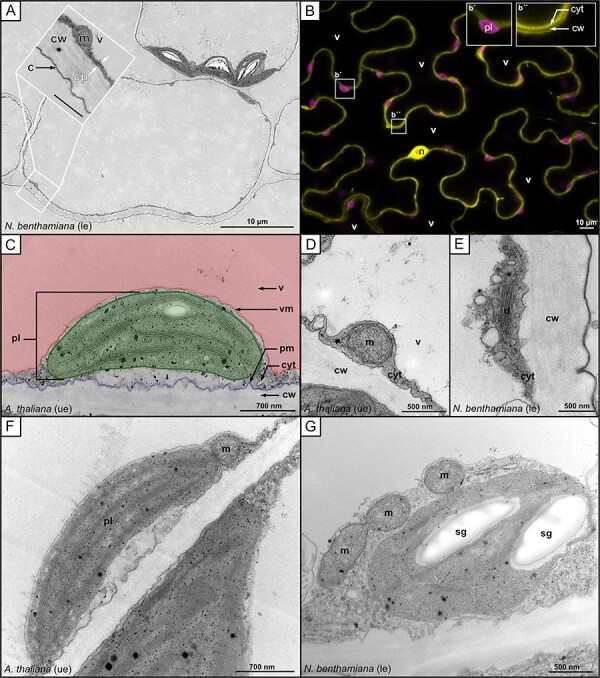
TEM and fluorescence images showing organelles in the cortical cytoplasm. (A) TEM image of a cross section through a leaf cell of the lower epidermis of *N. benthamiana*. The cytoplasm is visible as a thin dark gray lining along the inside of the lighter gray cell wall. The inset provides a detailed view of the cortical cytoplasm and the cell wall. (B) Confocal laser scanning microscopy image of a lower leaf epidermal cell of *N. benthamiana* expressing eGFP, which accumulates in the cytoplasm and nucleoplasm. Inset b´ shows a chloroplast as highlighted by its chlorophyll-A autofluorescence. Inset b´´ shows cytoplasmic GFP fluorescence of two cells separated by the non-fluorescent cell wall. (C–G) TEM images of (C) a plastid, (D) a mitochondria, (E) a dictyosome and (F) a plastid with one or (G) three mitochondria in close proximity to it in an area of the cell with enriched cytoplasm. v = vacuole, vm = vacuole membrane, pm = plasma membrane, cw = cell wall, c = cuticle, cyt/cp= cytoplasm, m = mitochondria, pl = plastid, sg = starch granule, d = dictyosome, n = nucleus, ue = upper epidermis, le = lower epidermis.

**Fig. 2:**
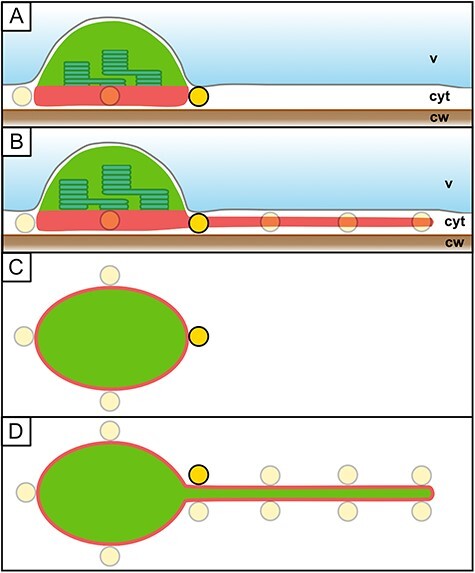
The spatial potential for plastid interactions in a quasi-2D cytoplasm. Plastids in the cortical cytoplasm (cyt) are pressed between the vacuole (v) and the cell wall (cw). (A) In the absence of stromule extension, only a minor fraction of the plastid’s surface area is available for interactions with other organelles (yellow circle) residing in the cortical cytoplasmic layer (interactive surface area = red line and red shading). (B) Forming a stromule, reaching into the cytoplasmic layer, increases the exposed surface area, allowing for more interactions. (C, D) Due to the observed constellation of plastids, stromules and other organelles in the thin cortical cytoplasmic layer (see **Fig. 1A****–****G**), an epidermal pavement cell can be projected into a 2D space for modeling approaches.

In order to understand these spatial constraints, and how plastids and other organelles are embedded in the cytoplasm of the cell, we performed transmission electron microscopy (TEM) and confocal laser scanning microscopy on pavement cells in the leaf epidermis of wild-type *Nicotiana benthamiana* and *A. thaliana* plants, as well as on transgenic *N. benthamiana* cells. The images shown in **Fig. 1** demonstrate that, as in the hypocotyl, these cells also host a large central vacuole that presses the cytoplasm with its content and the plasma membrane against the inner side of the cell wall, creating a very thin layer of cytoplasm (**Fig. 1A, Bb´´**). As a consequence, the largest portion of the cell lumen is occupied by the central vacuole (**Fig. 1A, B and Bb´´**). All other compartments reside within the thin cytoplasmic layer (**Fig. 1C****–****F**). As illustrated by TEM and confocal laser scanning fluorescence microscopy, many membrane-bound compartments are wider than the typical cytoplasmic layer, including plastids **(Fig. 1Bb, C**), mitochondria (**Fig. 1D**) and dictyosomes (**Fig. 1E**). As a consequence, these compartments expose only a small part of their surface toward the cytoplasmic layer (**Fig. 1C-F**), which represents the part of the compartment’s surface that is the easiest to access for compartment–compartment interactions (**Fig. 2A, B**). For the large plastids, the surface they expose to the cortical cytoplasmic layer is especially small relative to the rest of their surface area. Thus, for the interaction of a mitochondrion with a plastid, only the small area of the plastid surface, which is exposed to the cytoplasmic layer, is available, as depicted in the image shown in **Fig. 1F**. The cytoplasm ‘accumulates’ in only a few areas within an epidermal cell, providing a thicker layer in which organelles can ‘pile up’ on top of each other, making it possible for interactions to occur with all parts of the organelle surface. These areas typically form at the end of the epidermis pavement cell lobes (**Fig. 1G**) and around the nucleus, which resides in a ‘cytoplasmic pocket’ (**Fig. 1B, G**). Only in rare cases have we observed organelles such as mitochondria and peroxisomes ‘climbing’ the much larger plastid body, gaining access to the plastid membrane exposed to the vacuolar membrane. Taken together, these observations in epidermal pavement cells demonstrate that membrane-bound compartments are predominantly limited to interactions with the plastid body in the area defined by the thin cytoplasmic layer and not with its entire surface (**Fig. 2A**), in the quasi-2D ‘flatland’ of the cytoplasm (Abbott 2008). Therefore, we concluded that for modeling approaches, the interactions of plastids with their environment can be projected into a 2D space and still be representative (**Fig. 2C, D**).

### Physical properties of stromules extending from flattened plastids into the thin layer of the cortical cytoplasm

Following the finding that epidermal cells represent a quasi-2D environment for organelle interactions, we aimed to quantify the physical behavior of plastids and stromules within this quasi-2D plane. These data formed the foundation for later calculations.

#### *Stromule formation can be induced via inoculation with* Agrobacterium tumefaciens.

**Fig. 3:**
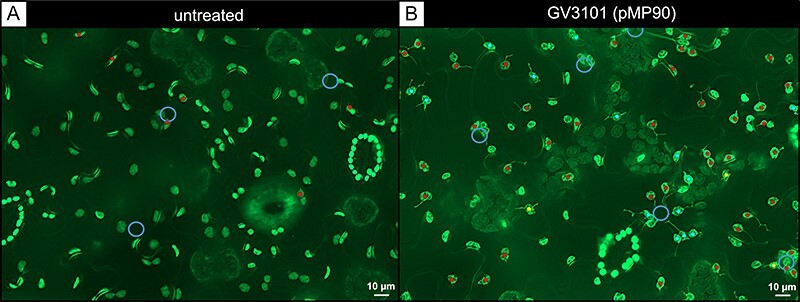
The extended depth of field fluorescence microscopy images of cells under control and stromule-inducing conditions. Images show lower epidermis cells of *N. benthamiana* transgenic line FNR:eGFP#7-25 in untreated (A) and treated (B) leaf areas. A strong induction of stromule formation is visible 3 d post inoculation with *A. tumefaciens* GV3101 (pMP90) at an OD_600_ of 0.8. Blue circles = position of nuclei; dots = plastids with stromules, red = 1, cyan = 2, yellow = 3.

While stromule levels are relatively low under typical growth conditions (unchallenged), they are strongly induced when plants face biotic stress. To better understand the advantage of stromules for a cell, it is of benefit to compare cells with basal stromule levels to those where stromules are induced. A suitable model system for such an approach is the treatment of *N. benthamiana* lower leaf epidermis with the laboratory strain of *A. tumefaciens* GV3101 (pMP90) (**Fig. 3A, B**). It is known from our previous work that stromule formation is triggered within 2–3 d post inoculation with higher optical densities of this strain (Erickson et al. 2014). In this case, stromules are specifically triggered by the activity of bacteria-secreted trans-zeatin, which significantly alters the physiology of cells within the infiltration spot, leading to the accumulation of soluble sugars and starch (Erickson et al. 2014). After 2 d, cells of inoculated leaf spots harbor a high number of stromules (>50% of plastids have one or more stromules) in contrast to untreated areas (<10%) (Erickson et al. 2014). Therefore, we decided to use GV3101 (pMP90)-treated tissue as a model for the quantitative analysis of plastids, stromules and cells. Quantification of stromule frequency (SF%) resulted in an average SF% of 11.6% in untreated tissue and an average SF% of 50.6% in treated tissue, confirming the stromule-inducing effect of GV3101 (pMP90).

#### The cortical cytoplasm of the epidermis cells is around 100 nm thick.

**Fig. 4:**
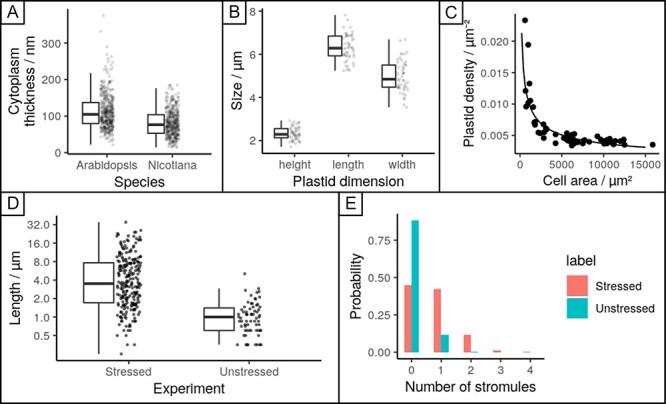
Quantitative measurements of cytoplasm, plastid and stromule-related statistics. (A) cytoplasmic thickness measured in TEM images of upper leaf epidermis cells of *A. thaliana* and lower leaf epidermis of *N. benthamiana*. (B) plastid dimensions length-width-height, measured in epifluorescence images. (C) Plastid density as measured in epifluorescence images as a function of cell size; curve gives a power-law fit. (D) Boxplot of stromule length measurements in epifluorescence images (only unbranched stromules of plastids with a single stromule considered) in unstressed and stressed samples. Boxplots show median, interquartile range, minimum and maximum. (E) Proportion of plastids with different numbers of stromules in unstressed and stressed cases. Number of samples: 4A *n* = 405 for both, 4B *n* = 50, 4C *n* = 55, 4D *n* = 235 for stressed and *n* = 67 for unstressed, 4E *n* = 978 for stressed and *n* = 1,152 for unstressed.

Before we started to look at the different stromule characteristics, we wanted to understand how thin the cytoplasm in our chosen model system (lower leaf epidermis cells of *N. benthamiana*) really is. Additionally, we wanted to see if our assumption is also true for another model species and therefore included *Arabidopsis thaliana* epidermis cells in our measurements. We quantified the thickness of the cytoplasm from the TEM images, finding that cytoplasm thickness in both *A. thaliana* upper leaf epidermis cells and *N. benthamiana* lower leaf epidermis cells was around 100 nm on average and exceeded 300 nm only in very rare cases (**Fig. 4A**). TEM and epifluorescence images of unchallenged *N. benthamiana* lower epidermis cells show that plastids are pressed against the cell wall, appearing mostly oval-shaped when viewed from the side (pressed against the anticlinal cell wall) and rather circular when viewed from the top (pressed against the periclinal cell wall; **Fig. 1A, B, C, F** and **Fig. 3A, B**). Furthermore, we used epifluorescence images to quantify the 3D dimensions of plastids in this environment, finding average values of 6.3 µm length, 5.0 µm width and 2.3 µm height (**Fig. 4B**).

#### *The density of plastids in* N. benthamiana *is constant, but only in larger epidermis cells.*

We also measured the density of plastids in cells of different sizes (**Fig. 4C**). We found that plastids are more densely packed (up to around 0.014 µm^−2^) in smaller cell, and that plastid density decreases drastically toward an asymptotic value (around 0.004 µm^−2^) in larger cells. If these densities are interpreted as corresponding to the size of a ‘box’ of cytoplasm in which only one plastid may be present, they correspond to characteristic plastid–plastid separations of between 8 and 15 µm. The behavior of plastid density with cell size can be well fit (*R*^2^ = 0.8, **Supplementary Fig. S1A**) by a power law, though given the limited multiplicative range involved this should be interpreted cautiously (Stumpf and Porter 2012). In summary, the lower epidermis of *N. benthamiana* leaf is dominated by large cells with a rather low density of plastids. In numbers, this means that 8% of the pavement cell area is occupied by small cells with plastid densities >0.005 µm^−2^, while the remaining 92% is covered by rather large cells with consistently lower plastid densities (<0.005 µm^−2^).

#### In unchallenged and stressed cells, the majority of stromules are short.

Stromules are often found to vary substantially in length. To characterize this variability in our model systems, we next measured the length distribution of stromules in unchallenged and stressed cells (**Fig. 4D**). We found that stromule length can be described in both conditions by a log-normal distribution (**Supplementary Fig. S1B, C**). Stromule length under stress conditions had a median of 3.5 µm (quartiles 1.7 and 7.7 µm), a several-fold increase from that under unstressed conditions (median 1.0 µm and quartiles 0.6 and 1.4 µm; **Fig. 4D**). Log-normal distributions are characteristic of quantities arising from independent multiplicative increments (like the scaling of individual elements) and are also observed in other dynamic, microscopic extension phenomena in biology, from the spines of neurons (Loewenstein et al. 2011) to cytoskeletal strands (Husainy et al. 2010, Polizzi et al. 2021). In all these cases, including stromules, populations are dominated in numbers by short structures and longer structures are increasingly rare. While not a focus of this investigation, the mechanistic basis for this distribution could readily be, e.g. the ongoing extension of a stromule by independent events where a motor protein attaches and elongates the stromule, scaling the length by a multiplicative factor before detaching. This is in agreement with current models for stromule elongation in which motor proteins are assumed to attach to the plastid surface, pulling out stromules by progressive movement along the respective cytoskeletal element.

#### Stromule count distributions per plastid are consistent with independent stromule initiations.

**Fig. 4E** shows the distributions of stromule count per plastid in stressed and unstressed conditions. This behavior can be well fit by a Poisson distribution (**Supplementary Fig. S1D, E**), which is the expected behavior if plastid initiation events are independent. In other words, the presence of one stromule on a plastid does not influence the probability that another is extruded; extrusion occurs independently and randomly with a characteristic rate. This rate is, on average, 0.69 per plastid in stressed samples (**Fig. 4E**). The stromule number per plastid also increases markedly under stress. Across samples, the Poisson model has a range of mean values 0.57–0.88 per plastid in the stressed case and 0.09–0.18 (mean 0.12) per plastid in the unstressed case, corresponding to the rate of stromule initiation increasing by between 3- and 6-fold under stress.

### Increases in physical capacity for plastid interaction due to stromule geometry

Following the delineation of different parameters of stromules, plastids and cells, we next asked if mathematical modeling could test the impact that stromules and plastid distribution have on plastid interactions with their cellular surroundings. For this, we introduced two parameters that describe two aspects of how plastids might interact with the cytoplasm and other organelles.

#### ‘Interaction region’ and ‘plastid access’ describe the spatial capacity of a plastid for interactions.

We define the ‘interaction region’ of a plastid as the area of the planar region within a given distance *d* of the plastid’s boundary. We also define the ‘plastid access’ of a given cellular point as the extent to which that point is surrounded by a plastid: specifically, the proportion of the circumference of a circle of radius *d* drawn around the point that contains part of the plastid. The interaction region then describes how much of the quasi-2D cytoplasm is ‘available’ to a plastid for interactions. The plastid access at a point describes how much of the circumferential surface of a putative organelle at that point could interact with the plastid.

#### Stromule extension increases the plastid’s interaction region and plastid access.

**Fig. 5:**
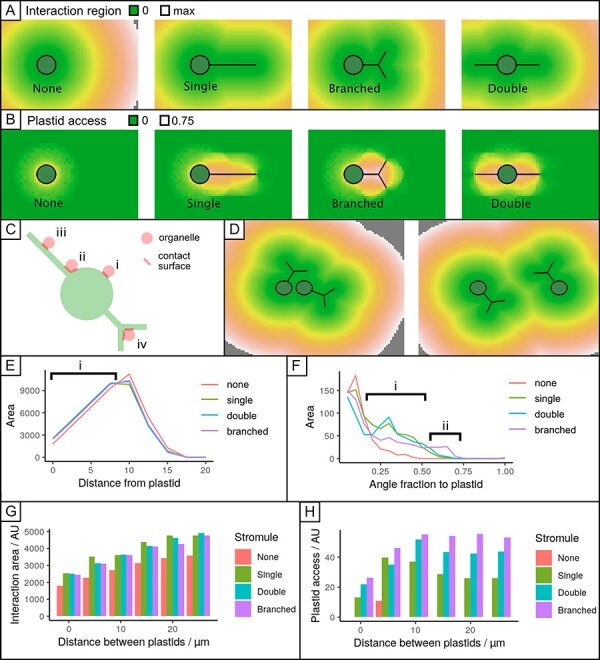
The interaction region and plastid access for different stromule geometries. (A) The interaction region of a plastid, plotted as the distance from a given point to the nearest part of the plastid. Distances range from 0 (within plastid) to ‘max’, the length scale of the model cell boundary. (B) Plastid access. A circle of radius *d* is drawn around each point. The proportion of the circumference for which a part of the plastid falls inside the circle is recorded. (C) Different arrangements of organelles and stromules. (i) Limited plastid access on the circumference of a circular plastid. (ii) Greater plastid access at the junction of a stromule. (iii) Limited axis along a stromule. (iv) High access at the branching point of a stromule. (D) Example of interaction region for a pair of plastids, changing with inter-plastid separation (left, 5 µm; right, 20 µm). (E) Distribution of cellular distances to the plastid for different stromule geometries (summary of (A)). In region (i), stromule branching supports a greater area of closer cytoplasm (cytoplasmic coverage) than for the case without stromules. The interaction area of plastid pairs with different separations (summary of (C)) is shown in (G). (F) Distribution of plastid access through the cell for different stromule geometries (summary of (B)). In region (i), stromule extension increases the amount of cytoplasm with high plastid access. In region (ii), branched stromules create some regions with very high plastid access. The behavior for plastid pairs with different separations is shown in (H).

We use a combination of mathematical modeling and simulation (Methods; **Supplementary Text; Fig. S5**) to describe how these quantities change with stromule length and geometry. We consider the ‘default’ case of a plastid without stromules and several different arrangements of stromules (preserving the total amount of membrane): a single long stromule, two shorter single stromules and a branched stromule (**Fig. 5A**; **Supplementary Fig. S2**). Keeping the total amount of plastid membrane constant in both cases, stromule extension substantially increases the plastid’s interaction region across different proximities *d* (**Fig. 5A, E**; **Supplementary Text**). Notably, the scale of this increase in interaction region is much more pronounced in 2D than would be the case in 3D (**Supplementary Text; Supplementary Fig. S3–4B**)—so the relative advantage of stromules for facilitating interaction is particularly high in the quasi-2D epidermal cytoplasm. A similar increase is found if the total plastid volume, rather than membrane, is kept constant (**Supplementary Text;**  **Supplementary Figs. S3–4A**). Taken together, the extension of a stromule introduces some regions of the cell (adjacent to the point where the plastid is extruded) that have high plastid access (**Fig. 5B, F**). A mitochondrion, for example, positioned at such a point will have part of its boundary adjacent to the main body of the plastid and another part of its boundary adjacent to the stromule, increasing the proportion of its boundary adjacent to the plastid (**Fig. 5C**).

#### Only well-spaced plastids gain full benefits from stromule formation.

We used this simulation approach to calculate the changes in these interaction region and plastid access when two plastids, separated by a given distance, were considered together (**Fig. 5D****–****H**). For plastids separated by ≤5 µm, comparatively little advantage is gained in either interaction region or plastid access. However, for higher separations, more substantial amplification occurs in both quantities so that the advantage of having two plastids separated by a distance of 10–15 µm is substantially greater. This length scale corresponds to the characteristic separation observed from the density measurements in **Fig. 4C**. The relatively higher advantage in a 2D geometry also holds here (Supplementary Text), highlighting the value of stromule extension for multiple plastids in the quasi-2D environment.

### Stromule branching increases the accessibility of a plastid to other cellular regions

#### Branched stromules increase the plastid access to cytoplasmic regions.

From the perspective of the interaction region alone, assuming a constant amount of membrane, branching is always at least slightly worse than extending a single long stromule (**Fig. 5B, F**). This is because the interaction region is ‘double counted’ in the region of a branch point: extending a long stromule will reach more cytoplasm than branching in the vicinity of an existing stromule. However, branched stromules achieve a different geometrical goal, which is increasing the plastid access of different cellular regions. A point near the junction of a branched stromule is more surrounded by plastid than one near a single continuous branch (**Fig. 5C**). For example, a mitochondrion positioned at the branch point would have most of its boundary within a small distance of the plastid, whereas one adjacent to a single stromule would have a limited amount of its boundary adjacent to the plastid. Correspondingly, branched stromules create regions of very high plastid access, where an organelle is mostly surrounded by plastid (**Fig. 5B, F**). To maximize the interaction region with a branch point, theory predicts that branching angles should be as close to 120° as possible (**Supplementary Text;**  **Supplementary Fig. S5**). This agrees with observations of branching angles between 100° and 140°, centered on 120° (Schattat et al. 2011b).

### Observed stromule behavior under stress enhances inter-organelle interaction capacity

Taken together, these theoretical and simulation results show that there is a trade-off for stromule structure—mirroring the ‘physical-social’ trade-offs observed in other organelle interaction behaviors (Chustecki et al. 2021). Single linear stromules maximize the region of the cell close to the plastid. However, stromule branching allows other organelles to maximize the amount of their perimeter in contact with the plastid. Branching at 120° provides a resolution to this trade-off, where the loss of proximal region is minimized while branching is supported. This theory then predicts that, depending on the relative weighting of two competing cellular priorities (maximizing a plastid’s capacity for interaction and maximizing an organelle’s capacity to interact with the plastid), stromules should favor either linear structures or those branching at 120°. These are the structures that are overwhelmingly most commonly observed in cells (**Fig. 3**; Schattat et al. 2011b), suggesting that biology adopts these optimal resolutions to meet the challenge of regulating organelle interactions in quasi-2D. At the same time, multiple plastids separated by distances over 5 µm amplify the advantages of these structures. Once again, the characteristic separation observed in cells (8–15 µm) fulfills this condition. Taken together, these observations support a picture where stress induces a response in stromule structure that supports increased inter-organelle communication. The dramatic increase in stromule initiation and length that we and others observed serves to increase the effective interaction area of a plastid. This effect is particularly pronounced in the quasi-2D cytoplasm in epidermal cells. The degree of branching of stromules controls a trade-off between the access of the plastid to other cellular components (our ‘interaction region’) and the access of other organelles to the plastid (our ‘plastid access’).

## Discussion

The impact of organelle shape on organelle function and cell viability is difficult to address experimentally. This is due in large part to the absence of morphology-specific mutants, especially in the case of plastid stromules. Thus, alternative approaches to evaluate the importance of organelle morphology are a promising avenue of inquiry. Here, we have combined electron microscopy and fluorescence imaging with mathematical and simulation modelling to reveal the physical details of organelle interaction in the cytoplasm with a focus on plastids, showing that the physical space in which colocalization must occur is thin to the point of being a quasi-2D ‘flatland’ (Abbott 2008). Given this restricted space, extension of (quasi-1D) stromules powerfully increases the proportion of the cell with which the plastid can interact: mathematical modeling demonstrates the relative amplitude of this effect in 2D versus less restricted 3D space. Branched stromules do not serve to increase this interaction region, but instead create regions of the cytoplasm more surrounded by plastid content so that organelles positioned there have amplified access to the plastid through their membrane. Stromule structures observed in plant cells constitute a range of optimal solutions to a trade-off between maximizing a plastid’s capacity for interaction and maximizing an organelle’s capacity to interact with the plastid.

### Insights into the mechanism of stromule initiation

Although in the past years fundamental progress has been made in understanding how individual stromules are established and shaped by the action of actin and microtubule-dependent processes (Kwok and Hanson 2003, Erickson et al. 2018, Kumar et al. 2018), it is still not known how the transition from a low stromule frequency (SF) state to an induced stromule state is achieved for a complete plastid population within a cell. The open questions are the following: how is the decision made which plastids will form a stromule? How is it decided if a plastid forms one or more stromules? How is the decision made to form a single straight stromule or if a stromule branch is initiated? Knowing the answer to these questions would provide important insights into how the plant cell integrates stress signals to mount a complex morphological response. Our analysis starts to answer some of these questions. The Poisson distribution of stromule initiation events among plastids (**Fig. 4E**) suggests that this process is random and independent in *N. benthamiana* lower epidermis cells and that possession of one stromule does not increase the probability of gaining a second. In other words, the extension of stromules is unlikely to occur via the concentrated, local activation of the required stromule building ‘machinery’, but rather evenly distributed and activated cellular components are expected to contribute. The random nature of stromule initiation events would also imply that stromules are equally likely to form from a given area of boundary whether it is part of the plastid or a preexisting stromule (hence forming a branch), but this remains to be tested in detail. So far, widespread stromule formation fits with previous observations that stromule extension in *N. benthamiana* relies on microtubules, which are found throughout the cell cortex (Erickson et al. 2018, Kumar et al. 2018). Based on this, we further hypothesize that in *N. benthamiana* the machinery responsible for a low SF state is similar to the one active when the cell transitions to a high stromule state [e.g. in response to GV3101 (pMP90) or as part of an effector-triggered immunity (ETI) response] and only its activity is increased. This seems to conflict with data from the upper epidermal pavement cells in *A. thaliana*, which suggests that in unchallenged tissue stromule formation occurs in close proximity to the nucleus and is likely actin driven (Erickson et al. 2017). However, in *N. benthamiana*, there is no study to demonstrate specific enrichment of stromules near the nucleus. Therefore, this discrepancy may depend on differences in the mechanisms of stromule formation employed by these two species or differences in mechanisms employed to maintain basal stromule levels in close proximity to the nucleus.

### The consequences of increased plastid access

Physical membrane–membrane contact between organelles is believed to increase the exchange of signaling molecules, lipids, and, in some cases, may also contribute to organelle positioning (Baillie et al. 2020). Our modeling of membrane accessibility tells us something about how interactions with smaller organelles might occur. Not only does the rounded surface of the plastid body have a limited contact surface, but movement by a smaller organelle in almost any direction will take it away from the plastid. This is in stark contrast to a plastid with a stromule, which has more contact surface, and since the smaller organelle is surrounded on several sides (especially in the case of a branched stromule), the likelihood of the smaller organelle losing contact with the plastid is less. Future considerations include how an increase in access might influence other factors, such as the duration of interactions in the dynamic cellular environment. For example, peroxisomes and mitochondria seem to ‘pile up’ against stromules when caught up in the cytoplasmic stream (Kwok and Hanson 2004a; Gunning 2005). The ‘catching’ of smaller organelles in these ‘high-access’ zones created by stromules could represent one means by which stromules not only increase the contact area but also increase the duration of interactions in a dynamic cellular environment. Here, the detailed time-lapse imaging of plastids together with other organelles will help to form the foundation for modeling in this direction.

### Balancing nuclear and cytosolic interactions of plastids

It is well known that plastid positioning is not random, but tightly controlled through associations with actin (Oikawa et al. 2008). Fluorescence images captured for this study show that plastids in epidermal pavement cells of unchallenged *N. benthamiana* leaves are evenly distributed within the cytosol (**Fig. 3A, B**). Additionally, the frequency of stromules is minimal in the absence of stress (**Fig. 1A**). It is readily accepted that when plants are confronted with stress stimuli, interactions and communication between organelles, cytosol and plasma membrane increase in importance (Medina-Puche and Lozano-Duran 2022). Recent attention has been paid to instances of plastid repositioning toward the nucleus, which occurs readily upon confrontation with biotic stress, such as exposure to pathogen-derived effectors and peptides that trigger immune responses (Caplan et al. 2008, 2015, Erickson et al. 2014, Erickson and Schattat 2018, Ding et al. 2019, Prautsch et al. 2023). In this context, plastid proximity to the nucleus is cited as important for the efficient transfer of retrograde signals such as H_2_O_2_ and proteins from plastids to nuclei (Caplan et al. 2008, 2015, Ding et al. 2019, Mullineaux et al. 2020). One would expect that, if plastid relocation to the nucleus is essential for responding to stress, most plastids would be found at the nucleus, especially during the integration of strong stress signals, such as those associated with ETI. However, at least in the context of our work, inoculation with GV3101 (pMP90) (**Fig. 3A, B**) and activation of ETI by XopQ expression result in only a subset of plastids relocating to the nucleus (Erickson et al. 2014, Prautsch et al. 2023), while the rest remain evenly spread (**Fig. 3B** and **Supplementary Fig. S6**). It is important to note that in the case of XopQ, it was shown that stromule formation and plastid clustering are independent processes and could serve different functions (Prautsch et al. 2023). While clustering of plastids at the nucleus decreases the distance that a retrograde signalling molecule must travel, this arrangement sacrifices the area of interaction of plastids with the cytoplasm. The fact that the even spacing of plastids is largely maintained during stress responses implies that cytosolic interactions (e.g. for maintaining biochemical performance), other than those with the nucleus (for signaling), are also of importance during these stresses. We calculated that the greatest gains in the overall interaction region actually occur when evenly spaced plastids extend stromules, which is what we observed in our experiments. It is possible that under such stress (where plastid clustering is common), despite the fact that there is little to be gained by forming a stromule on plastids in a cluster, they are subjected to the same stromule-inducing signal as more distant plastids, where the benefit of a stromule is much more substantial. It would seem that a balance between plastid repositioning and stromule extension (total access of cytoplasm) is struck to mount the appropriate response to a given external stimulus.

### Are stromules obsolete in densely packed mesophyll cells?

Emitting a stromule is not the only mode by which plastid access increases. Although we have only modeled a scenario with two plastids, this simple situation is enough to show that plastid access increases, even in the absence of a stromule, but only when plastids are close enough together (**Fig. 5**). This benefit becomes lost again when the plastid distance increases. In this case, organelles caught between two plastids gain access to both plastids at the same time. So, although we have found that stromules have a large impact on both interaction area and access in epidermal cells, which have low plastid densities, we would expect that mesophyll cells, which are densely packed with plastids, have higher average plastid access even without forming stromules and that the impact of stromule formation would be reduced. Interestingly, mesophyll cells have been reported to have fewer stromules than epidermal tissues (Hanson and Sattarzadeh 2008), supporting this idea. Experimental evidence is provided by a study conducted in *Nicotiana tabaccum* seedings, which showed that stromule length in hypocotyl cells, undergoing skotomorphogenesis, is negatively correlated with plastid density (Waters et al. 2004). This means that with increasing cell elongation and the resulting decrease in plastid density, the authors observed a strongly correlated increase in stromule length.

## Conclusions

In order to overcome the current experimental limitations for addressing the impact of plastid shape and shape change on organelle function and cellular organization, we combined the measurement of morphological parameters for plastids and stromules with mathematical modeling and simulation. This approach allowed us to look at open questions in this field from a new angle, lending new support for existing hypotheses and suggesting new explanations for observations that have not been explained up to now. Our study shows the potential of our cross-disciplinary approach for guiding hypothesis development and experimental design in this field. An understanding of how plastids and other organelles interact will increase general knowledge related to a wide range of biological processes that depend on metabolite, protein or lipid transfer to and from the plastid. The future challenge will be to test whether the predicted impact can be measured using physiological methods. Here, the development of tools to manipulate organelle shape and positioning will be crucial. It will also be important to consider that stromule formation is likely the result of multiple independent mechanisms (Erickson et al. 2018), which could have different consequences for plastid shape and function (Kohler and Hanson 2000, Waters et al. 2004).

## Materials and Methods

### Plant material and growth

In this study, wild-type *N. benthamiana* and a transgenic plant line were used, highlighting the plastid stroma with eGFP (enhanced green fluorescent protein; fused with FNR (ferredoxin NADP(H) oxidoreductase) transit peptide: eGFP #7-25) (for details, see Schattat et al. 2011b). The plants were grown in greenhouse chambers under long-day conditions, with 16 h of daylight (23°C), 8 h of darkness (19°C) and 55% relative humidity. The wild-type Col-0 plants of *A. thaliana* were grown in walk-in chambers under short-day conditions, with 8 h of light (21°C) and 16 h of darkness (17°C) and a relative humidity of 60%.

### Transmission Electron Microscopy

Leaf disks (3 mm) of *A. thaliana* (fully expanded leaves of 4-week-old plants) and *N. benthamiana* (the top-most fully expanded leaf of 5-week-old plants) were taken from the middle of the lamina and fixed with 3% (w/v) glutaraldehyde (Sigma, Taufkirchen, Germany) in 0.1 M sodium cacodylate buffer (SCB; pH 7.2) for 3 h at room temperature. After fixation, the samples were rinsed in SCB and postfixed with 1% (w/v) osmium tetroxide (Carl Roth, Karlsruhe, Germany) in SCB for 1 h at room temperature. Subsequently, the leaf segments were rinsed with water, dehydrated in a graded ethanol series [10%, 30%, 50%, 70% (containing 1% uranyl acetate), 70%, 90% and 2 × 100% for 30 min each], infiltrated with epoxy resin according to Spurr (1969) and polymerized at 70°C for 18 h. Ultrathin sections (70 nm) were made with an Ultracut E ultramicrotome (Leica, Wetzlar, Germany). Sections were transferred to Formvar-coated copper grids, post-stained with uranyl acetate and lead citrate with an EMSTAIN (Leica) and observed with a Libra 120 transmission electron microscope (Carl Zeiss Microscopy GmbH, Jena, Germany) operating at 120 kV. Images were taken using a dual-speed on-axis SSCCD camera (BM-2k-120; TRS, Moorenweis, Germany).

### Infiltration of Agrobacteria into *N. benthamiana* leaves

Standard transient expression protocols were used to carry out infiltrations. The bacterial ‘overnight’ culture was pelleted, resuspended and then left to incubate in infiltration media containing acetosyringone for approximately 2 h at room temperature. The infiltration medium, called Agrobacterium infiltration medium, consisted of 10 mM MgCl_2_, 5 mM MES pH 5.3 and 150 μM acetosyringone (Sigma-Aldrich, Deisenhofen, Germany). After incubation, the optical density of the suspension was adjusted to OD_600_ = 0.2 (*xopQ:mOrange2* expression) or OD_600_ = 0.8 (*A. tumefaciens* alone). A needleless syringe was used to infiltrate the *A. tumefaciens* strains into the intercostal fields of the lower side of the third or fourth leaf of 6–8-week-old *N. benthamiana* plants. Three days after infiltration, leaf disks were harvested from the infiltrated areas, and a non-infiltrated control and the lower leaf epidermis were observed using either an epifluorescence or a confocal microscope.

### Plasmids

The *xopQ:mOrange* expression construct was previously described (Erickson et al. 2018). For labeling the cytoplasm, untagged eGFP was expressed from pGGA2 (Schulze et al. 2012). Both pGGA11 and pGGA2 house the pBGWFS7 backbone with spectinomycin resistance cassette, allowing selection in *Agrobacterium*, with a 35S promoter and terminator.

### Confocal and epifluorescence imaging

Leaf disks were vacuum-infiltrated with tap water and mounted on glass slides. Subsequently, z-stacks of the lower epidermis were collected.

#### Epifluorescence imaging.

For image acquisition, an epifluorescence microscope (AxioObserver Z1) from Zeiss (Jena, Germany) equipped with an X-Cite fluorescence light source and an MRm monochrome camera (Zeiss) was used. GFP fluorescence was recorded using a 38 HE filter cube (Carl Zeiss AG, Jena, Germany). mOrange2 fluorescence was recorded using the 43 HE filter cube (Carl Zeiss AG). The microscope manufacturer’s software (ZenBlue, Zeiss, Germany) controlled image acquisition. All images were captured using a 40x/0.75 NA EC PLAN NEOFLUAR lens.

#### *Confocal Laser Scanning Microscopy imaging*.

For images used to calculate plastid density, as well as the stromule reaction toward *xopQ:mOrange2* expression, an LSM 780 from Zeiss, Germany, was used (excitation: Argon laser line 488 nm, HeliumNeon laser line 633 nm; MBS 488 nm/561 nm/633 nm; emission detection: eGFP 499–606 nm and chlorophyll-A 647–721 nm; lens: Plan-Apochromat 20x/0.8). The image used to demonstrate the thickness of the cytoplasm in **Fig. 1B** was captured with a Leica Stellaris CLSM (excitation: 474 nm; emission eGFP 489–537 nm, chlorophyll-A 671–697 nm; lens: HC PL APO CS2 40x/1.10 WATER).

### Image processing and quantification of stromule/plastid parameters

#### Stromule and plastid parameters.

For measuring stromule and plastid parameters, images of infiltrations with OD_600_ = 0.8 of GV3101 (pMP90) as well as images of untreated FNR:eGFP#7-25 plants were used. To obtain 2D extended depth of field images for quantification, single images of the z series of each channel were first exported into separate file folders and subsequently combined into single images using software and procedures described in Schattat and Klosgen (2009). For the quantification of stromule frequencies (SF%), we measured the proportion of plastids with at least one stromule (Erickson et al. 2014) with the help of the MTBCellCounter (Franke et al. 2015). Stromule length was measured by tracing stromules with the line tool and using the ‘skeletonise (2D/3D)’ as well as the ‘analyse skeleton’ function of Fiji (Schindelin et al. 2012). The number of plastids with different numbers of stromules was manually counted with the help of the MTBCellCounter (Franke et al. 2015). The proportions of the plastids were measured with the ‘free hand line’ tool in images of untreated leaves using Fiji (Schindelin et al. 2012). Plastid density measurements were made in maximum intensity projections along the z-axis of 3D z-stacks. The density of plastids is expressed as the number of plastids per unit of area for each analyzed cell. In order to obtain plastid density, cells were outlined in stacked bright field images (Schattat and Klosgen 2009). Outlines were used to measure the projected cell area in µm^2^, using Fiji (Schindelin et al. 2012). Superimposed outlines were used to count plastids in each outlined cell. Both measurements were used to calculate plastid density.

#### Cytoplasm thickness.

The thickness of the cytoplasm was measured in ultrathin section TEM images by adding ‘line’ drawing elements connecting the vascular membrane (tonoplast) to the plasma membrane, using Fiji (Schindelin et al. 2012). A random grid was used to choose measuring spots, avoiding measurement across organelles. Analogously to the measurements of the length of the stromules, the generated lines were analyzed for length in batch.

### Mathematical and computational models

Mathematical modeling was based on the representation of plastid bodies and stromules as simplified geometric objects such as circles, rectangles and cylinders, and calculating regions adjacent to these objects as a function of their arrangement (**Supplementary Text**). Simulations were carried out by embedding a set of line segments representing the plastid circumference and stromules of various geometries in the x–y plane. The plane was then partitioned into pixels. The distance between each pixel and its nearest line segment was computed to construct the interaction region of a plastid. Raytracing was used to compute the plastid access of a pixel. A ray was extended at angle *θ* from the pixel of interest, and if the ray intersected a plastid line segment within a distance *D* of the pixel, an encounter at angle *θ* was recorded. *θ* was scanned from 0 to 2π radians in increments of 0.1, and the proportion of angles for which an encounter was reported is recorded.

The plastid bodies were assigned radii of 2 µm. Line segments corresponding to stromules had conserved length *l* = 10 µm, which could be partitioned as a single stromule of length *l*, two stromules of length *l*/2 extended from opposite sides of the plastid body or a branched stromule where a segment of length *l*/3 branches to two other length *l*/3 segments all at 120° angles. The angle of extension of the first stromule was randomly varied in different simulation instances. We simulated the cases of a single plastid and two plastids with their centers separated by horizontal (x) distances of 5–25 µm in 5 µm steps. The simulation was carried out using custom code written in C; this code and R implementations of the mathematical results, statistical analyses and plotting scripts for all the modeling are freely available at https://github.com/StochasticBiology/stromule-geometry. R libraries used were *ggplot2* (Wickham 2016), *ggforce* (Pedersen 2021), *gridExtra* (Auguie 2017), *readxl* (Wickham and Bryan 2023) and *svglite* (Wickham et al. 2023) for input and output.

## Supplementary Material

pcad098_Supp

## Data Availability

All simulation and analysis code and structural data are freely available at github.com/StochasticBiology/stromule-geometry.
