# Supplementary material for: Stromule Geometry Allows Optimal Spatial Regulation of Organelle Interactions in the Quasi-2D Cytoplasm
Source: Plant Cell Physiol. 2023 Sep 2;65(4):618–30. doi: 10.1093/pcp/pcad098 (PMC11094753; doi:10.1093/pcp/pcad098)
Supplement: pcad098_Supp [file pcad098_supp.zip › suppl_data/pcp-2023-e-00101-File007.pdf]

Supplementary Information

Statistics and model fits

Fig. 1 shows model fits for plastid density, stromule length, and stromule count distributions.

Simulation output

Fig. 2 shows further examples of simulation output for different plastid separations and stromule geometries.

Linear stromule extension

We picture a plastid as a sphere with initial radius r_0. A cylindrical stromule of radius ρ and length l can be extended from the surface of the sphere. There are two possible pictures we can consider: (i) total plastid surface area is conserved (constant amount of membrane); (ii) total plastid volume is conserved (constant amount of stroma). In each case, as stromule length l increases, the radius of the spherical part of the plastid will decrease, as membrane/stroma is 'donated' to the stromule. We write r for the radius of the spherical part.

The total surface area and volume of sphere and stromule are

$$A = 4\pi r^2 + 2\pi\rho l \quad (1)$$

$$V = \frac{4}{3}\pi r^3 + \pi\rho^2 l \quad (2)$$

Under (i), total area is conserved, so $A = 4\pi r_0^2$ and

$$r_{(i)} = \sqrt{r_0^2 - \frac{\rho l}{2}}. \quad (3)$$

Under (ii), total volume is conserved, so $V = \frac{4}{3}\pi r_0^3$ and

$$r_{(ii)} = \sqrt[3]{r_0^3 - \frac{3}{4}\rho^2 l}. \quad (4)$$

Reflecting the fact that sphere and stromule are embedded in a thin layer of cytosol, we will consider the cytosol as a quasi-2D plane (Fig. 3A-B). We are interested in $\mathcal{A}(d)$, the area of the plane that lies within a distance d outside the plastid.

From the labelled areas in Fig. 3A-B,

$$\mathcal{A}(d) = A_3 + A_4 + A_5 - A_6 \quad (5)$$

$$\simeq \pi((r+d)^2 - r^2) + 2dl + \frac{1}{2}\pi(d+\rho)^2 - 2d^2 \quad (6)$$

where some approximations have been made and the subtraction of A_6 is to avoid double counting in that region. We can insert Eqns. 3-4 and simplify to obtain

$$\mathcal{A}_{(i)}(d) = \pi d(d + \sqrt{4r_0^2 - 2l\rho}) + \frac{\pi}{2}(d+\rho)^2 + 2dl - 2d^2 \quad (7)$$

$$\mathcal{A}_{(ii)}(d) = \pi d(d + \sqrt[3]{8r_0^3 - 6l\rho^2}) + \frac{\pi}{2}(d+\rho)^2 + 2dl - 2d^2. \quad (8)$$

Taking some images from [1] as a guide, we estimate as characteristic values $r_0 = 2\mu m$ (plastid radius), $l = 10\mu m$ (stromule length), $\rho = 0.25\mu m$ (stromule radius). Then Fig. 4A demonstrates that even a moderate stromule length dramatically increases the area of the cytoplasmic plane within a given distance of the plastid.

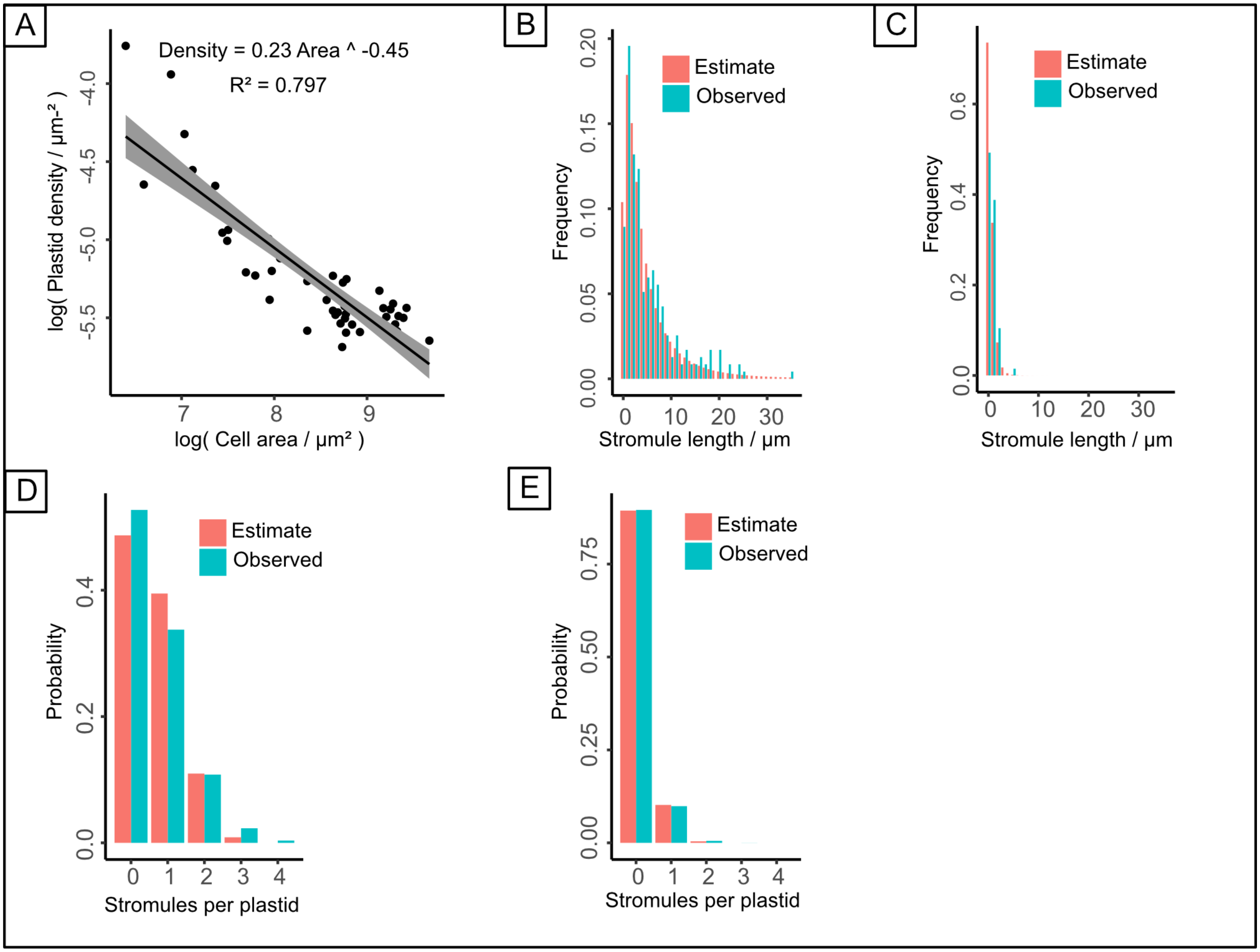

Figure 1: **Model fits for plastid and stromule statistics.** (A) Power-law fit relating plastid density to cell area. (B) Estimated log-normal distribution fitting observed stromule lengths in stressed samples. (C) Estimated log-normal distribution fitting observed stromule lengths in unstressed samples. (D) Estimated Poisson distribution fitting stromule counts per plastid in stressed samples. (E) Estimated Poisson distribution fitting stromule counts per plastid in an unstressed samples.

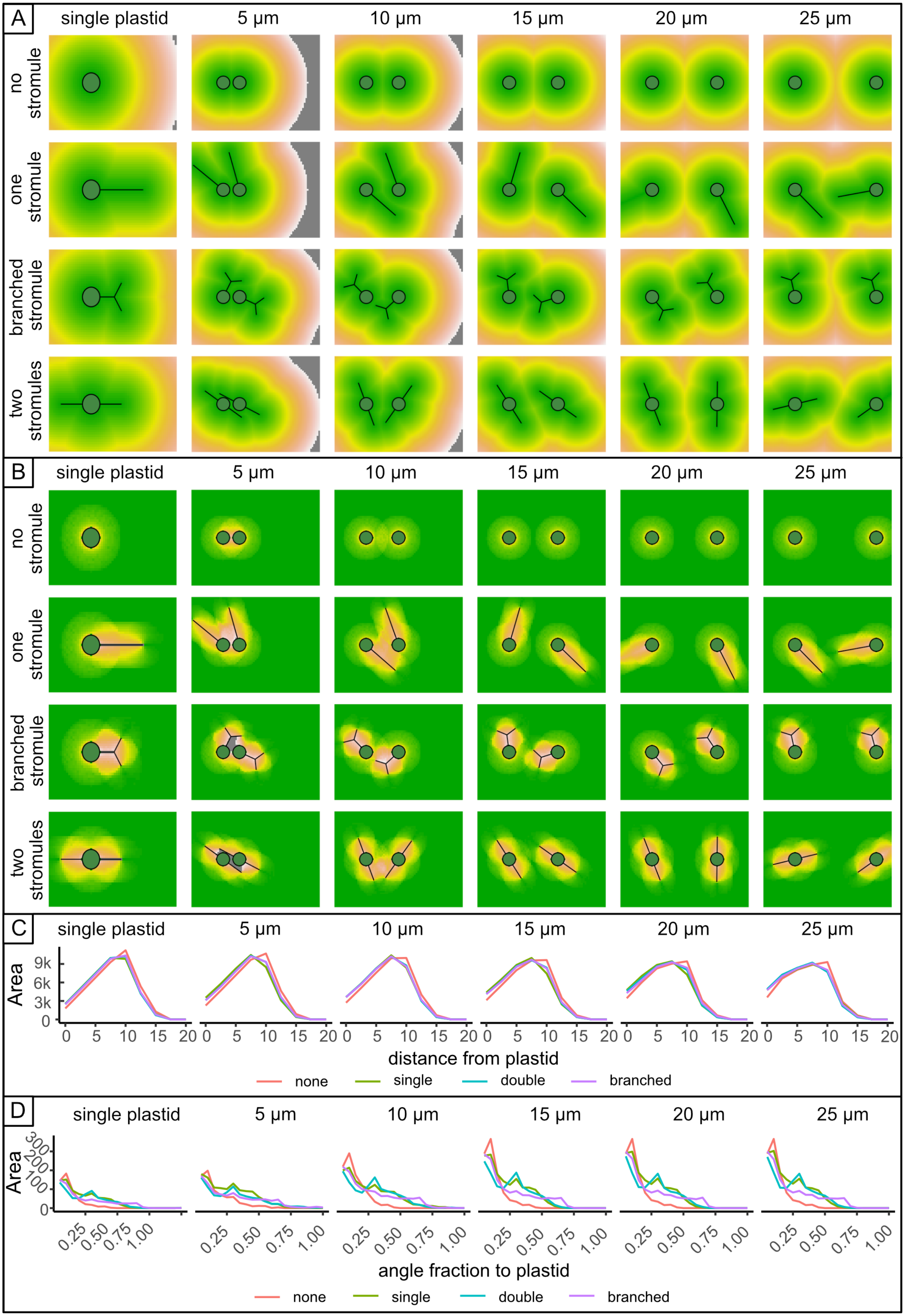

Figure 2: **Simulation outputs.** (A) Interaction region and (B) plastid access for (rows) different stromule structures and (columns) single plastids, then pairs of plastids separates by $5, 10, 15, 20, 25\mu m$. (C) Interaction region and (D) plastid access quantified from these simulations as in the main text; different panels give different plastid separations (0, single plastid).

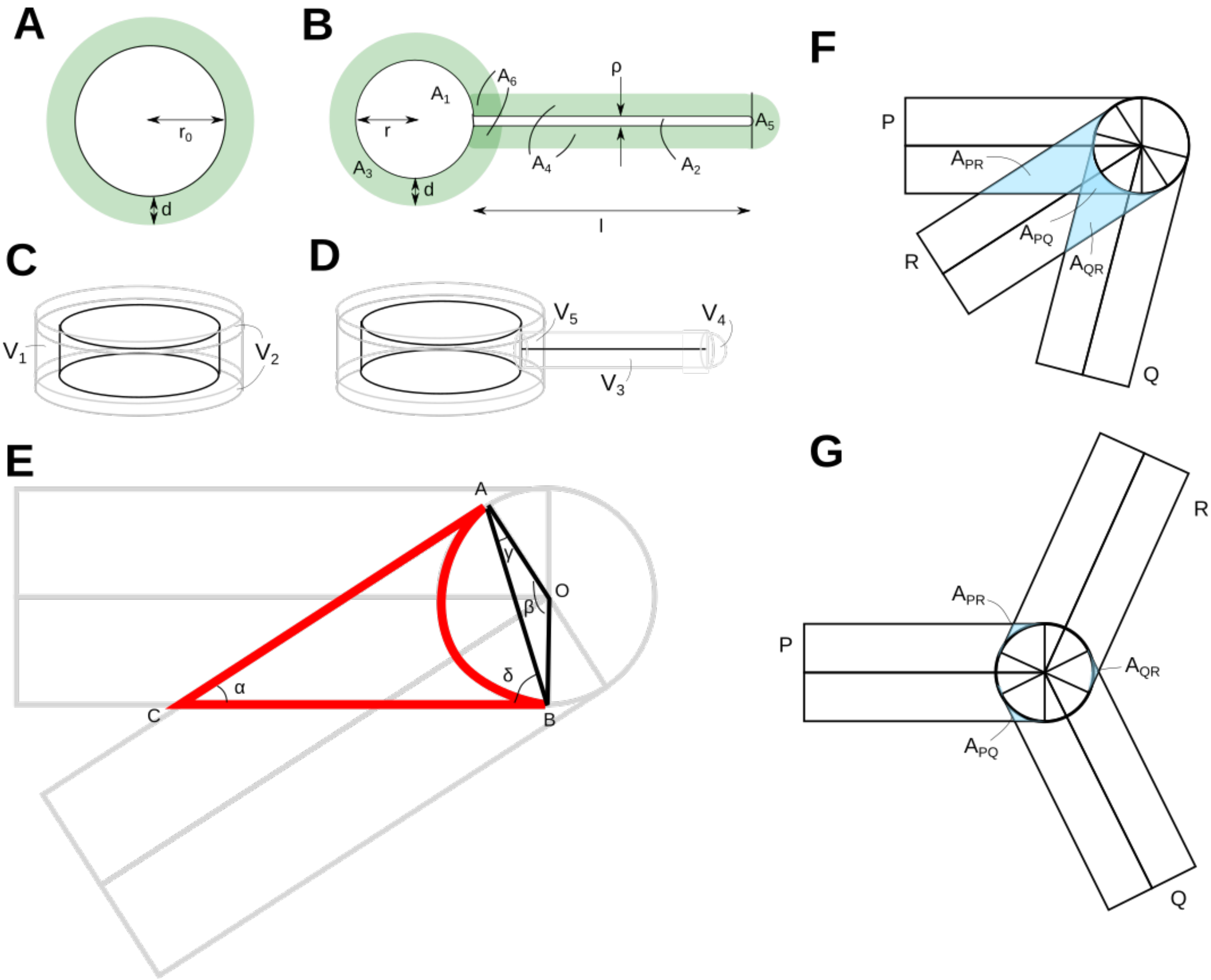

Figure 3: **Geometric models for stromules.** (A-D) Shells surrounding linear stromule extension model, in 2D with (A) no stromule and (B) extended stromule; and in 3D with (C) no stromule and (D) extended stromule. (E) Overlap region of shells around two stromule segments. (F) Overlapping regions around a branch point where one segment falls 'between' two others; (G) overlapping regions around a branch point where one segment falls 'outside' two others.

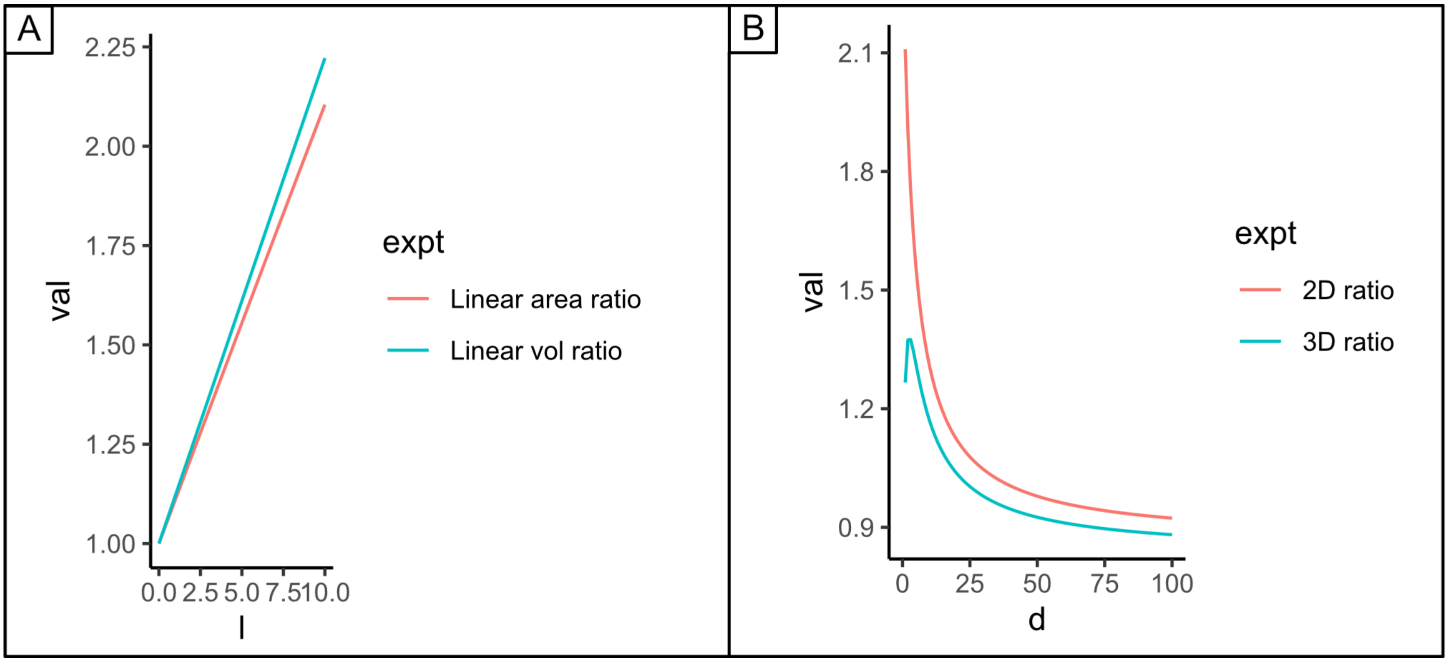

Figure 4: **Advantages of stromules.** (A) Increase of cytoplasmic area within a distance of 1 μm of the plastid as stromule length l increases, plotted relative to the area without a stromule. Lines show the cases when membrane area or internal volume are conserved. (B) Size of cytoplasmic region within a distance d of the plastid when a 10μm stromule is extended, relative to the no-stromule case. Relative increase is greater in 2D (area) than in 3D (volume).

Simplified geometry in 2D vs 3D

Given that the stromule radius is rather smaller than the other length scales, we can simplify this picture further. We consider the $\rho \to 0$ limit, where the stromule is a line with negligible width. We picture the body of the plastid as a circle, then compute the area of a shell around the plastid within distance d:

$$\mathcal{A}_{no} = \underbrace{\pi(r_0 + d)^2 - \pi r_0^2}_{A_3} \tag{9}$$

$$\mathcal{A}_{yes} = \underbrace{\pi(r + d)^2 - \pi r^2}_{A_3} - \underbrace{2d^2}_{A_6} + \underbrace{2ld}_{A_4} + \underbrace{(\pi/2)d^2}_{A_5} \tag{10}$$

In 3D we can picture the body of the plastid as a cylinder with radius r_0 and height h (Fig. 3C-D). The volume of a shall around the plastid within distance d is

$$\mathcal{V}_{no} = \underbrace{\pi(r_0 + d)^2 h - \pi r_0^2 h}_{V_1} + \underbrace{2\pi(r_0 + d)^2 d}_{V_2} \tag{11}$$

$$\mathcal{V}_{yes} = \underbrace{\pi(r + d)^2 h - \pi r^2 h}_{V_2} + \underbrace{2\pi(r + d)^2 d}_{V_2} + \underbrace{\pi d^2(l - d)}_{V_3 - V_5} + \underbrace{(2\pi/3)d^3}_{V_4} \tag{12}$$

Fig. 4B shows the relative difference in cytoplasmic region size within distance d of the plastid in the yes-stromule and no-stromule cases. In 2D, the relative increase in area is always higher than the relative increase in volume in 3D, especially for low d.

Branching angles

When a stromule changes angle or branches, the shell regions surrounding different segments of its structure may overlap. Fig. 3E shows an illustration of this, where an acute angle between stromule segments leads to the width-d shells of the two segments overlapping in the highlighted region. This overlap 'double counts' the corresponding region, so that overall less space is contained within a distance d of the plastid.

To compute how much space is lost in this way, we first compute the area of the highlighted region in Fig. 3E as a function of α, the angle between the two stromule segments.

Triangle OAB has base AB and height $d \sin \gamma$. Triangle ABC has base AB and height $\frac{1}{2} AB \frac{\tan \delta}{2}$. Quadrilateral $OACB$ thus has area $\frac{1}{2} ABd \sin \gamma + \frac{1}{2}(AB)^2 \frac{\tan \delta}{2}$.

Circle sector $\triangledown OAB$ has area $\pi d^2 \beta/(2\pi) = \beta d^2/2$. So the region of interest has area

$$\mathcal{A} = \mathcal{A}_{OACB} - \mathcal{A}_{\triangledown OAB} = \frac{1}{2} ABd \sin \gamma + \frac{1}{2}(AB)^2 \frac{\tan \delta}{2} - \frac{1}{2} \beta d^2 \tag{13}$$

As length $OA = d$, $AB/\sin \beta = d/\sin \gamma$, for $AB = d \sin \beta / \sin \gamma$.

As angles $\angle OAC$ and $\angle OBC$ are right angles, $\beta = \pi - \alpha$. By internal angles of triangles, $\gamma = (\pi - \beta)/2 = \alpha/2$, $\delta = (\pi - \alpha)/2$. Hence $AB = d \sin \alpha / \sin \alpha/2$ and overall

$$\mathcal{A} = \frac{1}{2} d^2 \frac{\sin \alpha}{\sin \alpha/2} \sin \alpha/2 + \frac{1}{2} d^2 \frac{\sin^2 \alpha}{\sin^2 \alpha/2} \frac{1}{2} \tan \frac{\pi - \alpha}{2} - \frac{1}{2}(\pi - \alpha) d^2 \tag{14}$$

$$= \frac{1}{2} d^2 \left(\sin \alpha + \frac{1}{2} \frac{\sin^2 \alpha}{\sin^2 \alpha/2} \tan \frac{\pi - \alpha}{2} - \pi + \alpha \right) \tag{15}$$

Now in the case of a branch point with three stromule segments P, Q, R incident on a branch point, there are two cases that require consideration. Without loss of generality, label P and Q as the segments with the largest angular separation. Then R can either fall 'between' or 'outside' the angle formed by P and Q (Figs. 3F and 3G respectively). In the 'between' case, the total overlap area is $A_{PR} + A_{QR} - A_{PQ}$, where the overlap of overlaps is subtracted to avoid double counting. In the 'outside' case, the total overlap area is the sum $A_{PR} + A_{QR} + A_{PQ}$. Then, treating the incident angle of P as the zero reference angle, we have

$$\mathcal{A} = A(\alpha_R) + A(\alpha_Q - \alpha_R) - A(\alpha_Q) \text{ if } \alpha_R < \alpha_Q \tag{16}$$

$$\mathcal{A} = A(\alpha_R) + A(\alpha_R - \alpha_Q) + A(\alpha_Q) \text{ if } \alpha_R > \alpha_Q. \tag{17}$$

Fig. 5 shows this overall overlap area as a function of the two angles, with a minimum at $\alpha_Q = 2\pi/3$ and $\alpha_R = 4\pi/3$ (120° separation).

References

[1] Maureen R Hanson and Kevin M Hines. Stromules: probing formation and function. *Plant physiology*, 176(1):128–137, 2018.

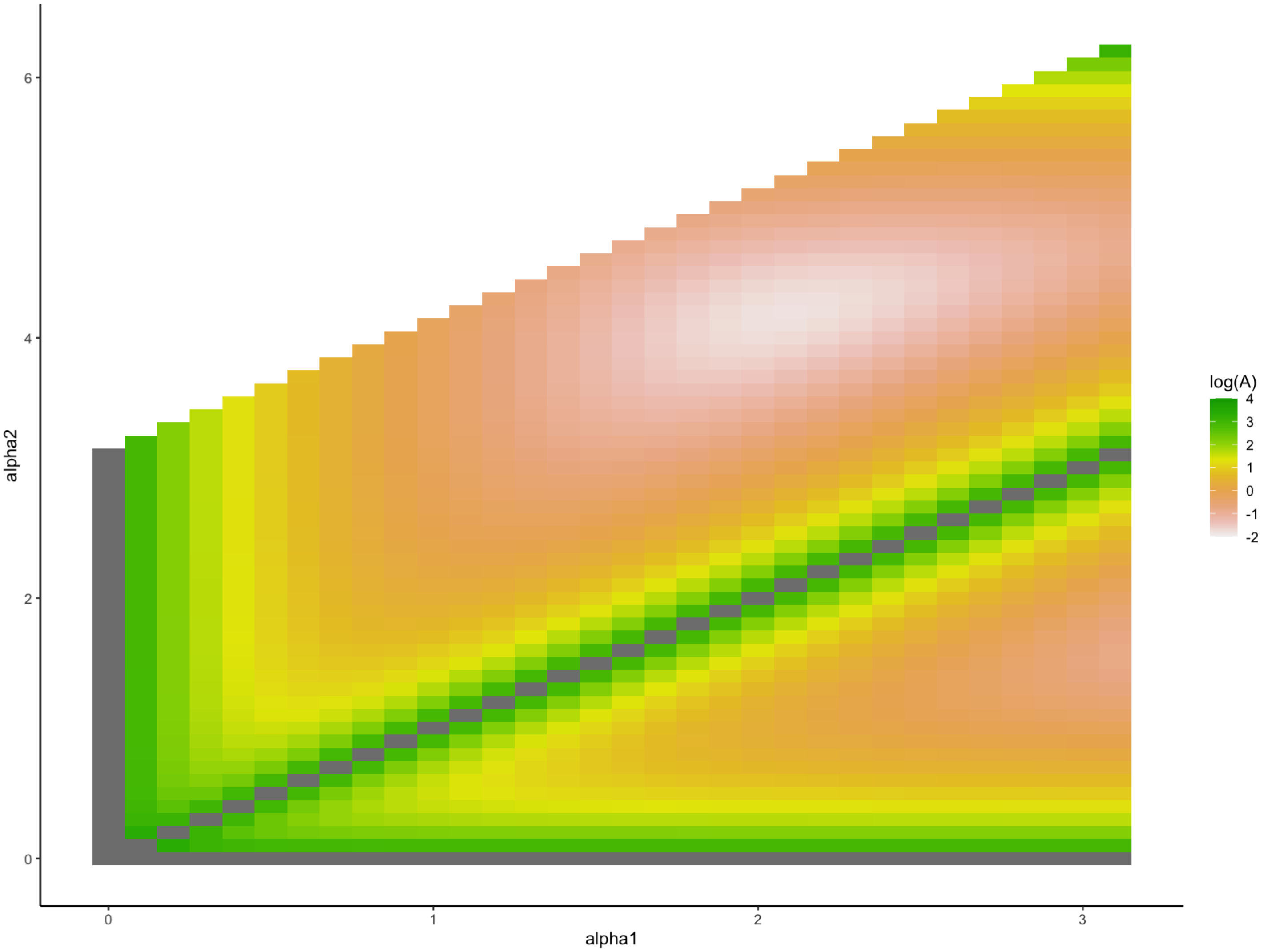

Figure 5: **Optimal branching angles.** Overlap area as a function of angles α_1 and α_2 in a stromule junction (logarithmic scale). Overlap area is minimised when angles are $2\pi/3 \simeq 2.1$ and $4\pi/3 \simeq 4.2$. A secondary minimum occurs at angles $\pi \simeq 3.1$ and $\pi/2 \simeq 1.6$. When angles are equal, or either are zero, stromule segments overlap perfectly and the overlap area diverges. Given the symmetry of the system, results are plotted only for $\alpha_1 < \pi$ and $\alpha_2 < \alpha_1 + \pi$ (values outside these inequalities can be mapped to this plot by relabelling the segments and angles).

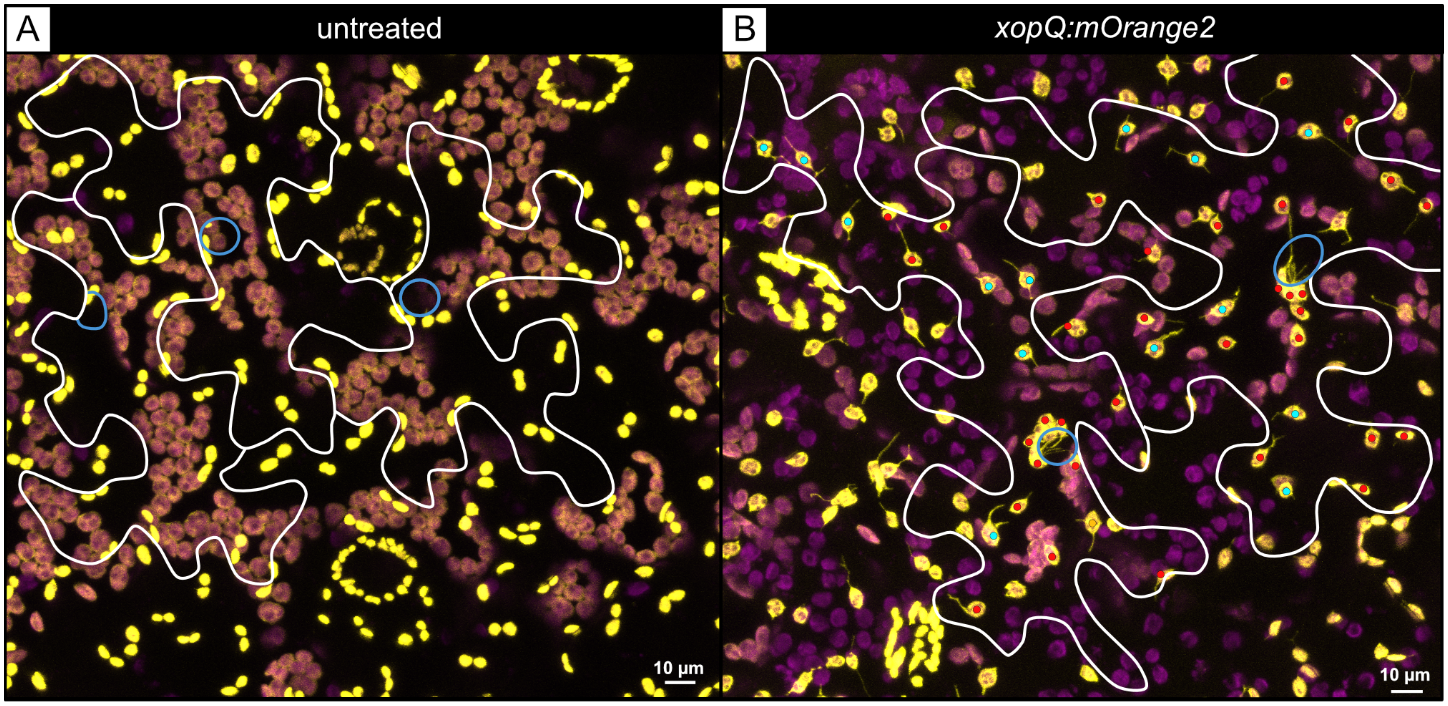

Figure 6: Maximum intensity projection along the z-axis of CLSM images capturing the lower epidermis cells of untreated (A) and treated (B) leaf sectors of a *N. benthamiana* transgenic line FNR:eGFP#7-25. The treated area was inoculated with *A. tumefaciens* cells mediating the expression of xopQ:mOrange2. White lines = cell outlines of pavement epidermis cells; blue lines = indicates the localisation of nuclei; coloured dots indicate plastids with stromules, red = 1, cyan = 2, yellow = 3.
